# Comparative study of large cell neuroendocrine carcinoma and small cell lung carcinoma in high-grade neuroendocrine tumors of the lung: a large population-based study

**DOI:** 10.7150/jca.33367

**Published:** 2019-07-10

**Authors:** Jian Wang, Ling Ye, Hui Cai, Meiling Jin

**Affiliations:** Department of Pulmonary Medicine, Zhongshan Hospital, Fudan University, Shanghai 200030, China.

**Keywords:** Large cell neuroendocrine carcinoma, small cell lung carcinoma, neuroendocrine tumors, high grade, lung

## Abstract

**Background**: In 2015, large cell neuroendocrine carcinoma (LCNEC) was removed from the large cell carcinoma group and classified with small cell lung carcinoma (SCLC) constituting two members of the high-grade neuroendocrine tumors (NETs) of the lung. However, the difference between high-grade LCNEC and SCLC in terms of clinicopathological characteristics and prognosis has not been fully understood owing to the rarity of LCNEC.

**Patients and methods**: Patients with high-grade LCNEC and SCLC at initial diagnosis between 2001 and 2014 were identified using the Surveillance, Epidemiology, and End Results (SEER) program database. Clinicopathological characteristics between high-grade LCNEC and SCLC were compared using the Pearson's chi-squared test or Fisher's exact test. Differences in overall survival (OS) and cancer-specific survival (CSS) were compared using the log-rank test, Cox models and propensity score matching (PSM) analysis.

**Results**: A total of 1223 patients with high-grade LCNEC and 18182 patients with high-grade SCLC were enrolled. To the best of our knowledge, this study involved the largest number of high-grade LCNEC patients to date, with respect to a comparison between high-grade LCNEC and high-grade SCLC patients. There were significant differences in age, sex, race, laterality, SEER stage, nodal status, surgery, radiation and chemotherapy, but not marital status, between high-grade LCNEC and SCLC patients. High-grade LCNEC patients had a better OS and CSS than high-grade SCLC patients. Subgroup analysis also confirmed the better prognosis of the high-grade LCNEC patients in the regional stage, distant stage and surgery subgroups. However, no significant difference in prognosis was observed between the two non-surgery subgroups, which was confirmed using PSM analysis. Furthermore, high-grade LCNEC patients showed different metastatic patterns to high-grade SCLC patients.

**Conclusion**: These results suggested that high-grade LCNEC and high-grade SCLC were different histological types, and that a detailed classification for high-grade NETs of the lung was needed.

## Introduction

Neuroendocrine tumors (NETs) of the lung are a special subtype of lung cancer. According to the 2015 World Health Organization (WHO) Classification of Lung Tumors, large cell neuroendocrine carcinoma (LCNEC) was removed from the large cell carcinoma and grouped together with typical carcinoid (TC), atypical carcinoid (AC) and small cell lung carcinoma (SCLC) within the NETs of the lung for the first time 1. Owing to the poorly differentiated features, LCNEC and SCLC were classified as the high-grade NETs of the lung, compared with low-grade TC and intermediate-grade AC. LCNEC was a rare histologic type of lung cancer with an incidence of approximately 3 %, while SCLC was a common histologic type of lung cancer accounting for 15-20 % of all lung cancers 2, 3. Compared with carcinoid, LCNEC and SCLC had higher mitotic rates, more necrosis and poorer prognosis, and could even manifest combined with other lung cancer types 4. Although LCNEC and SCLC shared several similar histologic and clinical features, questions remained as to whether it was reasonable to classify LCNEC and SCLC within the same category.

Owing to the rarity of LCNEC, only a limited number of studies have compared the clinicopathological characteristics and survival outcomes between LCNEC and SCLC. To the best of our knowledge, only two large population-based studies have been reported to date. Varlotto et al. 5 identified 1211 LCNEC, 35304 SCLC and 8295 other large cell carcinoma (OLC) in the Surveillance, Epidemiology, and End Results (SEER) registries (2001-2007), and reported that LCNEC presented more similarities to OLC than to SCLC in regard to clinicopathological characteristics and survival outcomes in patients undergoing surgery. However, Derks et al. 6 extracted the data relating to 952 LCNEC, 11844 SCLC, 19633 squamous cell carcinoma and 24253 adenocarcinoma from the Netherlands Cancer Registry (2003-2012) and found that stage IV LCNEC exhibited a similar metastatic pattern and survival rate to SCLC, while the clinical features of early-stage LCNEC resembled those of squamous cell carcinoma and adenocarcinoma.

In other studies, Asamura et al. 7 enrolled 366 surgically resected pulmonary NET patients (141 LCNEC and 113 SCLC) and found that LCNEC patients had a similar prognosis to SCLC patients. Similarly, another two independent studies revealed that no prognostic difference was identified between surgically resected LCNEC and SCLC patients 8, 9. These findings differed from those reported by Varlotto et al. 5 Further, all of the four small population-based studies showed that advanced LCNEC patients benefited from SCLC-based chemotherapy, rather than SCLC-based chemotherapy plus non-small cell lung cancer (NSCLC)-based chemotherapy, and showed similar survival outcomes to those found in advanced SCLC 10-13. However, Niho et al. 14 reported that advanced LCNEC patients receiving combination chemotherapy with irinotecan and cisplatin presented a poorer prognosis than advanced SCLC patients. Thus, according to these contradictory data, it is hard to conclude that LCNEC and SCLC exhibit the same clinical features, prognosis and treatment strategies.

In our study, we obtained the clinicopathological and prognostic data of patients with high-grade LCNEC (n=1223) and high-grade SCLC (n=18182) from the SEER program (2001-2014), which is a large population-based database supported by the American National Cancer Institute. To the best of our knowledge, this study identified the largest number of high-grade LCNEC and SCLC patients, compared with previous studies. The clinicopathological characteristics and survival outcomes between LCNEC and SCLC were compared to improve our understanding of high-grade NETs of the lung.

## Patients and methods

### Data source and ethics statement

SEER provided cancer incidence statistics from population-based cancer registries covering approximately 34.6 % of the U.S. population. The specialized database "Incidence-SEER 18 Regs Custom Data (with additional treatment fields) Nov 2016 Sub (1973-2014 varying)" was applied to extract data using the SEER*Stat software, version 8.3.5 (released on 6 March 2018). Informed consent was not required in this study because identifying information on individual patients was excluded. These data are publicly available and we obtained access to the SEER data by signing the SEER Research Data Agreement. This study was approved by the Ethical Committee and Institutional Review Board of Zhongshan Hospital, Fudan University. No personal identifying information is stored in SEER database.

### Patient selection

Patients diagnosed with histologically confirmed high-grade LCNEC and SCLC from 2001 to 2014 were enrolled in the study. The inclusion criteria used to identify eligible patients were as follows: (1) age at diagnosis ≥ 18 years; (2) primary tumor site was restricted to "Lung and Bronchus" (based on site recode ICD-O-3 [International Classification of Diseases for Oncology, Third Edition]/WHO 2008); (3) pathological confirmation of LCNEC (ICD-O-3 8013/3) and SCLC (ICD-O-3 8041/3); (4) only one primary tumor; (5) high-grade tumor (grade III or IV); (6) the diagnosis was not confirmed by autopsy or death certificate; and (7) complete survival data. Cases of LCNEC and SCLC with low, intermediate or unknown grade were excluded because of the possible confusion with carcinoids.

### Covariates and outcomes

The covariates included age, sex, race, marital status, laterality, tumor size, SEER stage, nodal status, surgery, radiation, chemotherapy and distant metastasis (liver, bone and brain). Age was stratified into three groups: < 60, 60-79, and ≥ 80 years. Tumor size (cm) was categorized as follows: ≤ 3, > 3 and ≤ 5, > 5 and ≤ 7, and > 7 cm. SEER stage was classified into localized, regional, distant, and unknown according to the SEER program. Radiation and chemotherapy were categorized as “yes” or “no/unknown”. No site-specific metastasis data is available before 2010 in the SEER database.

Overall survival (OS) and cancer-specific survival (CSS) were identified as the primary survival outcomes in the study. OS was identified from diagnosis to death due to any cause, while CSS was calculated from diagnosis to death due to lung cancer. The cutoff date for follow-up was December 31, 2014. Any patient who died from other causes before this cutoff date, or who was alive on the date of last contact, was censored.

### Statistical analysis

Clinicopathological characteristics between high-grade LCNEC and SCLC were compared using the Pearson's chi-squared test or Fisher's exact test. The Kaplan-Meier method was used to generate survival curves. Differences between these curves were analyzed using the log-rank test. Univariate and multivariate Cox proportional hazard models were applied to identify risk factors for CSS, and the hazard ratios (HRs) and 95% CIs were reported. In the non-surgery subgroup, a propensity score matching (PSM) method to 1:1 match LCNEC with SCLC patients was applied to eliminate the difference in baseline characteristics across groups. The matching covariates included sex, race, laterality, tumor size, nodal status, and chemotherapy. The PSM method was undertaken using the psmatch2 module in Stata v14.0 (StataCorp, College Station, TX, USA). However, in the surgery subgroup, the number of patients in each of the two groups was almost the same, and the PSM method was not applicable. All statistical analyses were performed using SPSS v20.0 software (IBM, Armonk, NY, USA). A two-tailed *P* value of < 0.05 was considered to be statistically significant.

## Results

### Clinicopathological characteristics of high-grade LCNEC and SCLC

A total of 19405 patients with high-grade NETs of the lung were enrolled in the study, including 1223 patients with high-grade LCNEC and 18182 patients with high-grade SCLC. The demographic and clinical characteristics of these patients are described in Table 1. All covariates, except marital status, showed a significant difference between high-grade LCNEC and SCLC cases. Compared to high-grade SCLC patients, high-grade LCNEC patients were younger (< 60 years: 28.4 % *vs.* 24.9 %; ≥ 80 years: 36.4 % *vs.* 41.3 %; *P* = 0.001), predominantly male (55.6 % *vs.* 49.7 %; *P* < 0.001) and predominantly black (11.9 % *vs.* 8.6 %; *P* < 0.001), with a smaller tumor size (≤ 3 cm: 33.8 % *vs.* 18.3 %; *P* < 0.001 ) and a higher proportion of patients receiving surgery (49.0 % *vs.* 3.6 %; *P* < 0.001). However, high-grade SCLC patients presented a higher prevalence of distant metastasis according to SEER stage (67.5 % *vs.* 41.2 %; *P* < 0.001) and nodal metastasis (71.1 % *vs.* 48.7 %; *P* < 0.001), and had a higher proportion of patients receiving radiation (46.2 % *vs.* 34.5 %; *P* < 0.001) and chemotherapy (68.8 % *vs.* 51.3 %; *P* < 0.001), than high-grade LCNEC patients. These data suggested that high-grade LCNEC patients presented distinctly different clinicopathological characteristics to high-grade SCLC patients.

### Comparison of survival between high-grade LCNEC and SCLC

**Figure 1:**
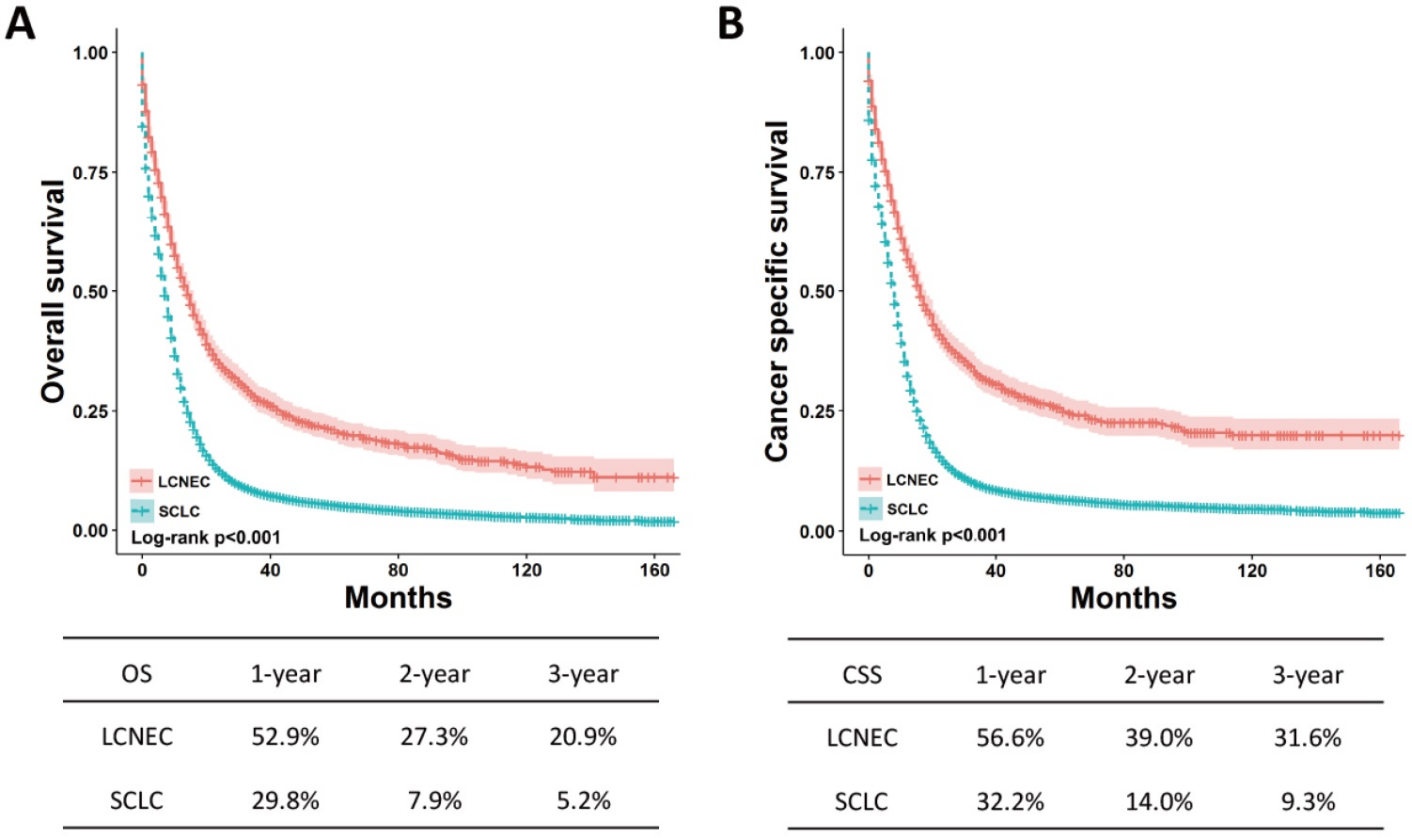
** OS and CSS for the high-grade LCNEC and SCLC patients using** Kaplan-Meier analysis and log-rank test. (A) OS: LCNEC *vs.* SCLC, *P* < 0.001; (B) CSS: LCNEC *vs.* SCLC, *P* < 0.001. Abbreviations: LCNEC, large cell neuroendocrine carcinoma; SCLC, small cell lung carcinoma; OS, overall survival; CSS, cancer-specific survival.

The OS and CSS between high-grade LCNEC and SCLC patients can be illustrated by Kaplan-Meier plots (Fig. 1). High-grade LCNEC patients exhibited a better OS and CSS than high-grade SCLC patients (*P* < 0.001). The one-, two- and three-year OS and CSS for high-grade LCNEC patients were also higher than those for high-grade SCLC patients. To further identify the prognostic factors involved in OS and CSS, univariate and multivariate Cox proportional hazard models were used to analyze the data. In the univariate analysis, histological type, age, sex, marital status, laterality, tumor size, SEER stage, nodal status, surgery, radiation and chemotherapy were found to be significantly associated with OS and CSS (*P* < 0.05) (Table S1). Next, these covariates were included and adjusted within the multivariate analysis (Table 2). As expected, all these covariates remained the prognostic factors for OS and CSS. Specifically, the following were poor prognostic factors for OS and CSS: older male, not married, increased tumor size, advanced SEER stage, nodal metastasis and no treatment (surgery, radiation or chemotherapy). Moreover, the multivariate analysis showed that high-grade SCLC patients presented a worse OS and CSS than high-grade LCNEC patients (OS: HR = 1.25, 95% CI 1.16-1.35, *P* < 0.001; CSS: HR = 1.26, 95% CI 1.16-1.37,* P* < 0.001).

### Subgroup analysis with SEER stage and surgery

**Figure 2:**
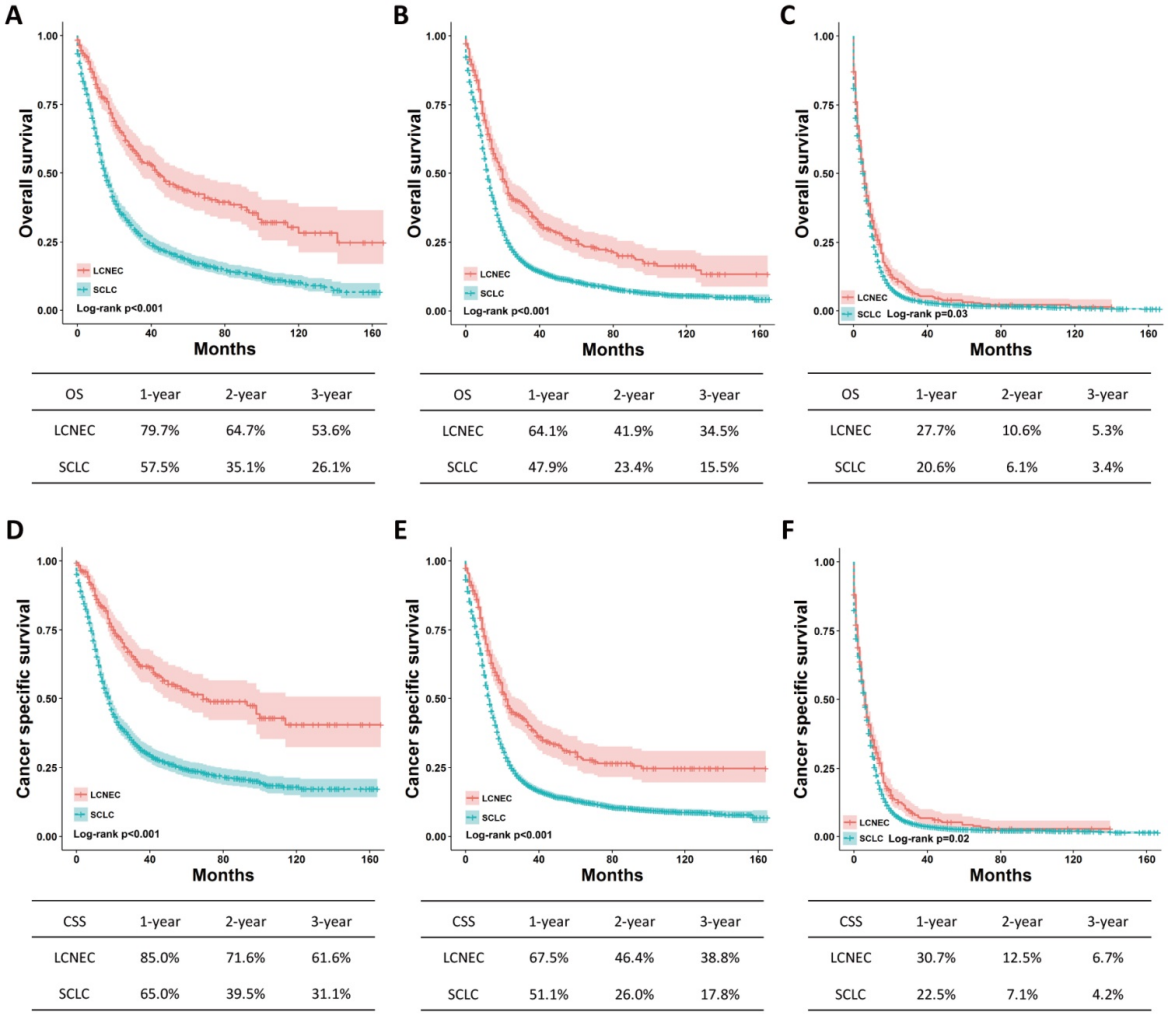
** OS and CSS for the high-grade LCNEC and SCLC patients in the SEER stage subgroup using** Kaplan-Meier analysis and log-rank test. (A) OS in localized stage subgroup: LCNEC vs. SCLC, *P* < 0.001; (B) OS in regional stage subgroup: LCNEC vs. SCLC, *P* < 0.001; (C) OS in distant stage subgroup: LCNEC vs. SCLC, *P* = 0.03; (D) CSS in localized stage subgroup: LCNEC vs. SCLC, *P* < 0.001; (B) CSS in regional stage subgroup: LCNEC vs. SCLC, *P* < 0.001; (C) CSS in distant stage subgroup: LCNEC vs. SCLC, *P* = 0.02. Abbreviations: LCNEC, large cell neuroendocrine carcinoma; SCLC, small cell lung carcinoma; OS, overall survival; CSS, cancer-specific survival; SEER, Surveillance Epidemiology and End Results database.

**Figure 3:**
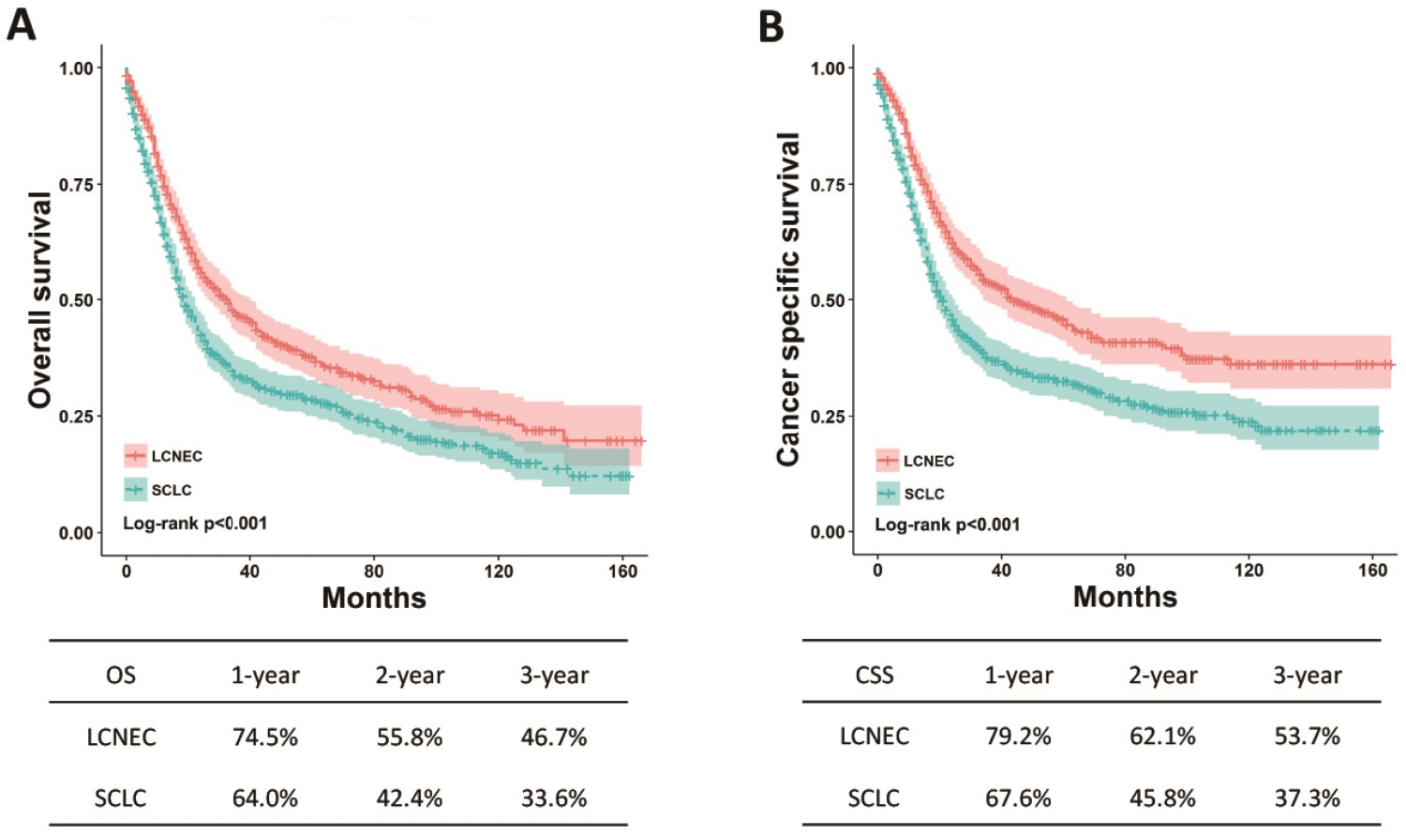
** OS and CSS for the high-grade LCNEC and SCLC patients in the surgery subgroup using** Kaplan-Meier analysis and log-rank test. (A) OS in subgroup treated with surgery: LCNEC vs. SCLC, *P* < 0.001; (B) CSS in subgroup treated with surgery: LCNEC vs. SCLC, *P* < 0.001. Abbreviations: LCNEC, large cell neuroendocrine carcinoma; SCLC, small cell lung carcinoma; OS, overall survival; CSS, cancer-specific survival.

The differences regarding OS and CSS between high-grade LCNEC and SCLC patients were further evaluated using stratified analysis based on SEER stage and surgery. The Kaplan-Meier plots showed that the high-grade LCNEC patients had a better OS and CSS than the high-grade SCLC patients in the localized, regional and distant subgroups (*P* < 0.05) (Fig. 2). In the subgroup with surgery, the high-grade LCNEC patients also presented a better OS and CSS than the high-grade SCLC patients (*P* < 0.05) (Fig. 3). However, no significant differences in OS and CSS between these two groups were identified in the non-surgery subgroup (Fig. S1). Furthermore, univariate and multivariate Cox proportional hazards analyses were used to address the HRs of these two histological types in different subgroups (Table 3). In the regional, distant and surgery subgroups, high-grade SCLC was found to be a risk prognostic factor for OS and CSS in our univariate and multivariate analysis. Although high-grade SCLC patients with localized stage had a poorer OS and CSS than high-grade LCNEC patients in our univariate analysis, no differences in OS and CSS were found between these two groups in our multivariate analysis. Furthermore, no differences in OS and CSS were observed in our univariate analysis of the non-surgery subgroup.

### Survival analysis in matched group

**Figure 4:**
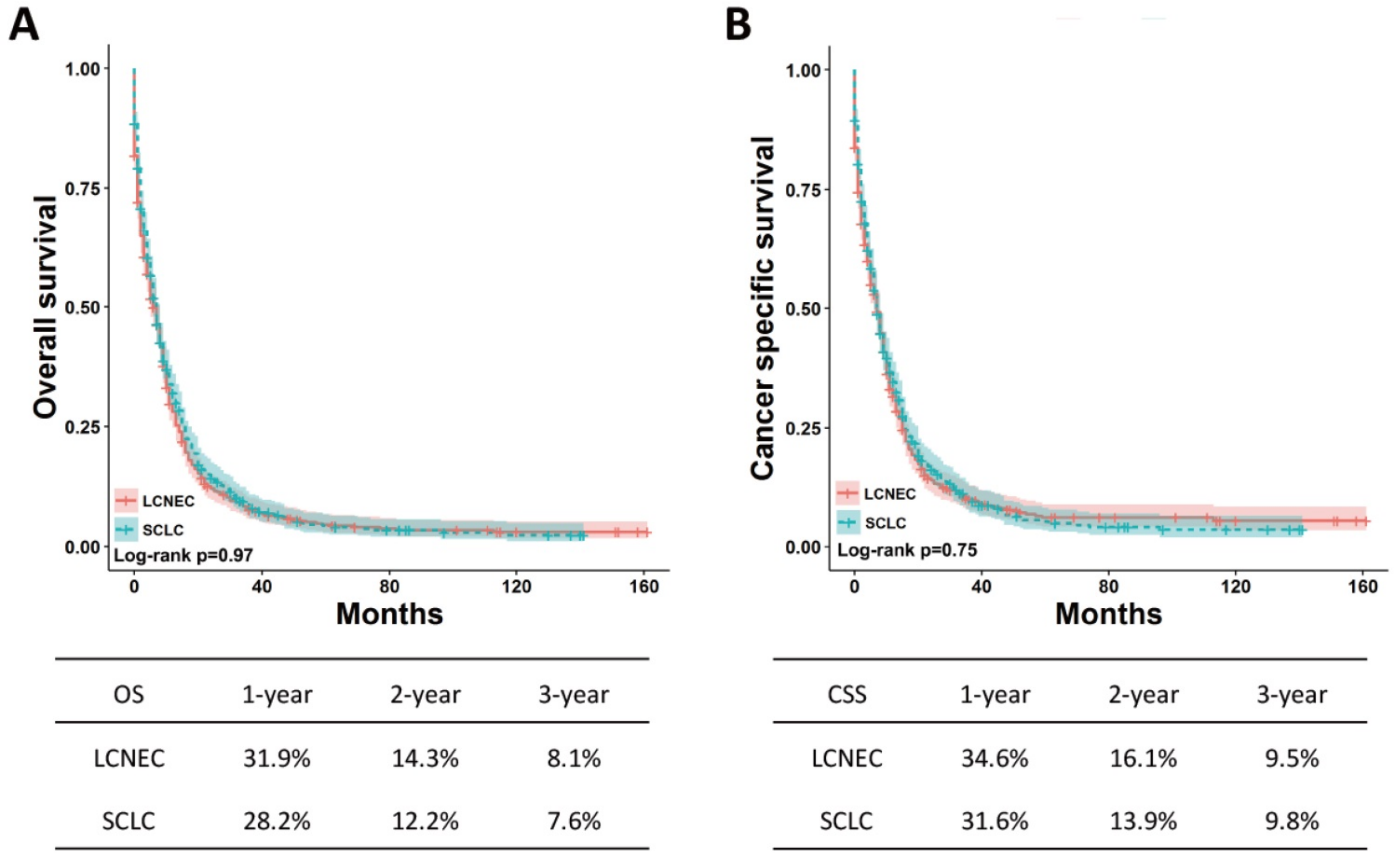
**OS and CSS for the high-grade LCNEC and SCLC patients without surgery in matched groups using** Kaplan-Meier analysis and log-rank test. (A) OS: LCNEC *vs.* SCLC, *P* = 0.97; (B) CSS: LCNEC *vs.* SCLC, *P* = 0.75**.** Abbreviations: LCNEC, large cell neuroendocrine carcinoma; SCLC, small cell lung carcinoma; OS, overall survival; CSS, cancer-specific survival.

To avoid the effect of confounding factors on OS and CSS between high-grade LCNEC and SCLC patients without surgery, the PSM method was applied to perform a 1:1 matched case-control analysis. A total of 1240 patients were enrolled for further analysis, including 620 high-grade LCNEC cases and 620 high-grade SCLC cases (Table 4). No significant difference in clinicopathological characteristics was found between these two groups. After matched analysis, the OS and CSS between the two groups remained the same (Fig. 4). These results suggested that the prognosis for the high-grade LCNEC patients without surgery was similar to that for the high-grade SCLC patients without surgery.

### Comparison of metastatic sites between high-grade LCNEC and SCLC patients

**Figure 5:**
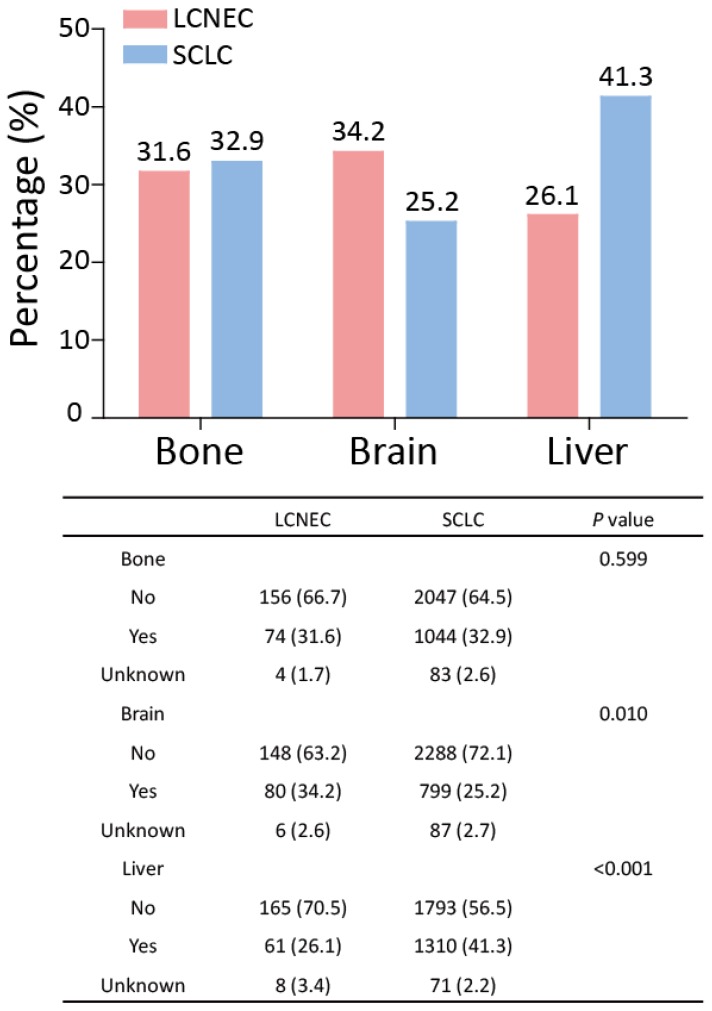
** Metastatic patterns for the high-grade LCNEC and SCLC patients with stage IV recorded between 2010 and 2014.** The differences in bone, brain and liver metastases between high-grade LCNEC and SCLC patients were analyzed using a Chi-square test. Abbreviations: LCNEC, large cell neuroendocrine carcinoma; SCLC, small cell lung carcinoma.

In the SEER database, the data for metastatic sites were available for patients who had been diagnosed since 2010. Thus, the data for stage IV high-grade LCNEC and SCLC patients recorded between 2010 and 2014 were extracted and further analyzed. Next, the percentages of stage IV high-grade LCNEC and SCLC patients with bone, brain, or liver metastasis were determined (Fig. 5). In high-grade LCNEC patients, brain metastasis was the favorite metastasis site, followed by bone and liver metastasis. In high-grade SCLC patients, liver metastasis was the most common metastasis site compared with bone and brain metastasis. Furthermore, the proportion of high-grade LCNEC patients, compared to the proportion of high-grade SCLC patients, differed significantly for brain and liver metastasis, but not for bone metastasis. Interestingly, high-grade LCNEC patients had significantly more brain metastasis than high-grade SCLC patients (34.2% *vs.* 25.2%; *P* < 0.010), while high-grade SCLC patients showed significantly more liver metastasis than high-grade LCNEC patients (41.1%* vs.* 26.1%; *P* < 0.001). These results suggested that the high-grade LCNEC and SCLC patients exhibited different metastatic patterns.

## Discussion

To the best of our knowledge, this study involved the largest number of high-grade LCNEC patients, with respect to a comparison between high-grade LCNEC patients and high-grade SCLC patients. Our findings indicated significant differences in clinicopathological characteristics between high-grade LCNEC and SCLC patients. High-grade LCNEC patients showed a better OS and CSS than high-grade SCLC patients and this remained true even after adjustment for other covariates in the multivariate analysis. The subgroup analysis also confirmed the better prognosis of high-grade LCNEC patients in the regional stage, distant stage and surgery subgroups. However, no significant difference in outcomes between high-grade LCNEC and SCLC was observed, once patients missed surgery, and this result was further confirmed by a PSM analysis. Furthermore, high-grade LCNEC patients had different frequencies and sites of distant metastasis compared with high-grade SCLC patients.

High-grade lung NETs accounted for 91.3 % of all high-grade NETs and displayed unique clinicopathological characteristics and outcomes compared with the remaining lung NETs (TCs and ACs) 15, 16. Since 2015, LCNEC has been grouped with SCLC to high-grade lung NETs for some similar features, but the difference between high-grade LCNEC and SCLC has not been fully recognized, mainly owing to the rarity of LCNEC. Several small comparative studies reported no significant differences in demographic and clinical characteristics between high-grade LCNEC and SCLC patients who underwent surgery 9, 17, 18. However, the small number of cases involved in these studies limited this conclusion. Fortunately, two large population-based studies provided more reliable evidences for the difference between high-grade LCNEC and SCLC. Varlotto et al. 5 used the SEER database (2001-2007) to establish that LCNEC patients present significantly different demographic and clinical characteristics compared with SCLC patients. LCNEC patients were more likely to be male and undergo surgery, while SCLC patients were found to be more likely to have advanced stage and nodal metastasis. Further, these differences still remained between LCNEC and SCLC among patients who received surgery without radiation. The shortcoming of Varlotto et al's study was to include LCNEC and SCLC patients of unknown grade, although the percentage of low- and intermediate-grade LCNEC and SCLC was less than 1 %. Derks et al. 6 analyzed the cases from the Netherlands Cancer Registry (2003-2012) and obtained similar results regarding the differences in sex, stage and nodal metastasis between high-grade LCNEC and SCLC. Moreover, they found that high-grade SCLC patients had larger tumors than high-grade LCNEC patients. In this study, we enrolled the largest number of high-grade LCNEC patients and excluded all patients with low, intermediate or unknown grade included in the SEER database (2001-2014). To improve the robustness of our analysis, we included more covariates of clinicopathological characteristics. Our results for the differences in sex, stage, tumor size, nodal metastasis, and surgery between high-grade LCNEC and SCLC were closer to those from the two large population-based studies. Furthermore, our findings indicated that high-grade LCNEC patients were younger and predominantly black, while high-grade SCLC patients were more likely to receive radiation and chemotherapy. Besides, we firstly analyzed the marital status and found a similar proportion of married/unmarried in high-grade LCNEC patients to that in SCLC patients.

The difference in prognosis between high-grade LCNEC and SCLC was not well-defined. Several retrospective studies showed a similar prognosis between high-grade LCNEC and SCLC patients who underwent surgical resection 7, 9, 18. However, using the Kaplan-Meier method, Varlotto et al. 5 showed that the OS and CSS for the high-grade LCNEC patients undergoing surgery without radiation were better than those for the high-grade SCLC patients. Isaka et al. 17 also found that stage IA LCNEC patients undergoing surgery had a better OS than SCLC patients. Interestingly, Derks et al. 6 found that early-stage high-grade LCNEC patients had a better OS than SCLC patients, but found no significant difference between LCNEC and SCLC in patients receiving surgery. Moreover, stage IV high-grade LCNEC patients had a worse OS than SCLC patients, but no significant difference was found between LCNEC and SCLC in patients treated with chemotherapy. Besides, Naidoo et al. 19 suggested that the OS of stage IV LCNEC resembles that of SCLC. In this study, we found that high-grade LCNEC patients had better OS and CSS than SCLC patients. Furthermore, our subgroup analysis showed that better OS and CSS were observed in high-grade LCNEC patients in the regional, distant, and surgery subgroups. However, in the non-surgery subgroup, no significant difference in OS and CSS was found between high-grade LCNEC and SCLC patients. These results were further confirmed by the multivariate analysis and PSM analysis.

Up to now, the therapeutic options for high-grade LCNEC have rarely been debated on account of its rarity. The National Cancer Control Network (NCCN) advised treating LCNEC according to the guidelines for non small-cell lung cancer, but clinicians have tended to use SCLC-based chemotherapy regimens for advanced LCNEC patients 19, 20. However, it was also difficult to decide whether LCNEC patients should be treated in the same way as SCLC patients. In the present study, data regarding the chemotherapy regimens for high-grade LCNEC and SCLC patients were not available in the SEER database, but some other evidence was found to assist with the clinical decision concerning high-grade LCNEC and SCLC. Our subgroup analysis showed that high-grade LCNEC patients could benefit much more from surgery treatment than high-grade SCLC patients. Once high-grade LCNEC patients had missed the opportunity for surgery, there was no difference in prognosis between the two groups. Thus, surgery is a more important therapeutic option for high-grade LCNEC patients than for high-grade SCLC patients.

For advanced LCNEC and SCLC patients, data regarding differences in the occurrence of distant metastasis has been limited. Derks et al. 6 firstly showed that LCNEC patients had fewer liver, and more brain metastasis than SCLC patients. Moreover, a high incidence of brain metastasis was observed in two small studies in LCNEC patients 19, 21. In our own study, we confirmed that high-grade LCNEC patients exhibited different metastatic patterns to high-grade SCLC patients. The brain was the most common metastatic site for high-grade LCNEC, while the liver was the most common site for high-grade SCLC.

This study has several limitations. The SEER database did not provide sufficient data on smoking history, self-reported information from patients, laboratory tests, imaging examination, chemotherapy regimens and even gene mutation examination, which reduced the significance of our results. We excluded those patients of unknown grade to avoid the confounders from carcinoids, but a wealth of information concerning high-grade LCNEC and SCLC was therefore not taken into account because fewer than 1 % of all the LCNEC and SCLC cases were diagnosed as grade 1 or 2 5. Furthermore, no data about distant metastasis for patients diagnosed before 2010 was available in the SEER database. This greatly reduced the amount of data available in terms of the number of high-grade LCNEC and SCLC patients for the comparative analysis of metastatic patterns. Besides, some missing data for tumor size in high-grade SCLC patients weakened the statistical difference between two groups.

In conclusion, high-grade LCNEC patients present different clinicopathological characteristics to high-grade SCLC patients. Compared with high-grade SCLC patients, high-grade LCNEC patients were found to have a better prognosis in the all stages, regional stage, distant stage and surgery cohorts. However, no significant difference in prognosis was found between high-grade LCNEC and SCLC in the non-surgery subgroup. Besides, high-grade LCNEC patients showed different metastatic patterns to high-grade SCLC patients. These findings provide strong evidence that high-grade LCNEC and high-grade SCLC are different histological types, and that a detailed classification for high-grade NETs of the lung is needed.

## Supplementary Material

Supplementary figure and table.

## Figures and Tables

**Table 1:** Clinicopathological characteristics of high-grade LCNEC and SCLC patients

	LCNEC N=1223 (%)	SCLC N=18182 (%)	*P* value^a^
**Age**			0.001
<60	347 (28.4)	4528 (24.9)	
60-79	431 (35.2)	6138 (33.8)	
≥80	445 (36.4)	7516 (41.3)	
**Sex**			<0.001
Female	543 (44.4)	9143 (50.3)	
Male	680 (55.6)	9039 (49.7)	
**Race**			<0.001
White	1026 (83.9)	16001 (88.0)	
Black	146 (11.9)	1572 (8.6)	
Others^b^	48 (3.9)	594 (3.3)	
Unknown	3 (0.2)	15 (0.8)	
**Marital status**			0.555
Married	637 (52.1)	9202 (50.6)	
Not married^c^	545 (44.6)	8393 (46.2)	
Unknown	41 (3.4)	587 (3.2)	
**Laterality**			0.019
Left	499 (40.8)	7380 (40.6)	
Right	684 (55.9)	9981 (54.9)	
Bilateral	16 (1.3)	176 (1.0)	
Unknown	24 (2.0)	645 (3.5)	
**SEER stage**			<0.001
Localized	316 (25.8)	1126 (6.2)	
Regional	390 (31.9)	4368 (24.0)	
Distant	504 (41.2)	12267 (67.5)	
Unknown	13 (1.1)	421 (2.3)	
**Tumor size (cm)**			<0.001
≤3	465 (38.0)	3329 (18.3)	
3-5	277 (22.6)	3243 (17.8)	
5-7	147 (12.0)	2195 (12.1)	
>7	142 (11.6)	2463 (13.5)	
Unknown	192 (15.7)	6952 (38.2)	
**Nodal status**			<0.001
No	563 (46.0)	2997 (16.5)	
Yes	595 (48.7)	12922 (71.1)	
Unknown	65 (5.3)	2263 (12.4)	
**Surgery**			<0.001
No	620 (50.7)	17374 (95.6)	
Yes	599 (49.0)	649 (3.6)	
Unknown	4 (0.3)	159 (0.9)	
**Radiation**			<0.001
No/ Unknown	801 (65.5)	9776 (53.8)	
Yes	422 (34.5)	8406 (46.2)	
**Chemotherapy**			<0.001
No/ Unknown	595 (48.7)	5674 (31.2)	
Yes	628 (51.3)	12508 (68.8)	

Abbreviations: LCNEC, large cell neuroendocrine carcinoma; SCLC, small cell lung carcinoma; SEER, Surveillance Epidemiology and End Results database. ^a^ *P* value between high-grade LCNEC and SCLC was calculated by chi-square test. ^b^ Others included American Indian/Alaskan native, and Asian/Pacific islander. ^c^ Not married included separated, single (never married), divorced, unmarried or domestic partner and widowed.

**Table 2:** Multivariate Cox proportional hazards regression model analysis of overall survival and cancer-special survival in high-grade LCNEC and SCLC patients

	Overall survival		Cancer-special survival	
	HR (95% CI)	*P* value	HR (95% CI)	*P* value
**Age**				
<60	Reference	—	Reference	—
60-79	1.15 (1.10-1.19)	<0.001	1.13 (1.08-1.17)	<0.001
≥80	1.43 (1.38-1.49)	<0.001	1.38 (1.33-1.44)	<0.001
**Sex**				
Female	Reference	—	Reference	—
Male	1.15 (1.12-1.19)	<0.001	1.13 (1.10-1.17)	<0.001
**Marital status**				
Married	Reference	—	Reference	—
Not married^b^	1.07 (1.04-1.10)	<0.001	1.05 (1.01-1.08)	0.005
Unknown	1.04 (0.95-1.13)	0.423	1.02 (0.94-1.12)	0.627
**Laterality**				
Left	Reference	—	Reference	—
Right	1.00 (0.97-1.03)	0.937	1.00 (0.97-1.04)	0.857
Bilateral	1.05 (0.91-1.22)	0.508	1.09 (0.94-1.26)	0.282
Unknown	0.82 (0.75-0.89)	<0.001	0.80 (0.73-0.87)	<0.001
**SEER stage**				
Localized	Reference	—	Reference	—
Regional	1.37 (1.27-1.48)	<0.001	1.48 (1.36-1.61)	<0.001
Distant	2.45 (2.28-2.65)	<0.001	2.72 (2.51-2.95)	<0.001
Unknown	1.02 (0.90-1.16)	0.708	1.10 (0.97-1.26)	0.138
**Tumor size (cm)**				
≤3	Reference	—	Reference	—
3-5	1.15 (1.09-1.21)	<0.001	1.17 (1.11-1.23)	<0.001
5-7	1.21 (1.14-1.28)	<0.001	1.23 (1.16-1.30)	<0.001
>7	1.25 (1.18-1.32)	<0.001	1.27 (1.20-1.34)	<0.001
Unknown	1.30 (1.25-1.36)	<0.001	1.34 (1.28-1.41)	<0.001
**Nodal status**				
No	Reference	—	Reference	—
Yes	1.22 (1.17-1.28)	<0.001	1.23 (1.17-1.29)	<0.001
Unknown	1.17 (1.10-1.24)	<0.001	1.19 (1.12-1.27)	<0.001
**Surgery**				
No	Reference	—	Reference	—
Yes	0.47 (0.43-0.51)	<0.001	0.45 (0.41-0.49)	<0.001
Unknown	0.83 (0.71-0.98)	0.025	0.81 (0.68-0.96)	0.016
**Radiation**				
No/ Unknown	Reference	—	Reference	—
Yes	0.70 (0.68-0.72)	<0.001	0.71 (0.68-0.73)	<0.001
**Chemotherapy**				
No/ Unknown	Reference	—	Reference	—
Yes	0.44 (0.42-0.46)	<0.001	0.44 (0.42-0.45)	<0.001
**Histological type**				
LCNEC	Reference	—	Reference	—
SCLC	1.25 (1.16-1.35)	<0.001	1.26 (1.16-1.37)	<0.001

Abbreviations: LCNEC, large cell neuroendocrine carcinoma; SCLC, small cell lung carcinoma; SEER, Surveillance Epidemiology and End Results database; HR, hazard ratio; CI, confidence interval. ^a^ Others included American Indian/Alaskan native, and Asian/Pacific islander. ^b^ Not married included separated, single (never married), divorced, unmarried or domestic partner and widowed.

**Table 3:** Subgroup analysis of high-grade LCNEC and SCLC with SEER stage and surgery.

	Overall survival				Cancer-special survival			
	Univariate analysis		Multivariate analysis		Univariate analysis		Multivariate analysis	
	HR (95% CI)	*P* value	HR (95% CI)	*P* value	HR (95% CI)	*P* value	HR (95% CI)	*P* value
**SEER stage**								
** Localized**								
LCNEC	Reference	—	Reference	—	Reference	—	Reference	—
SCLC	2.14 (1.81-2.53)	<0.001	1.19 (0.96-1.48)	0.120	2.36 (1.95-2.86)	<0.001	1.17 (0.91-1.51)	0.226
** Regional**								
LCNEC	Reference	—	Reference	—	Reference	—	Reference	—
SCLC	1.16 (1.06-1.28)	<0.001	1.33 (1.15-1.54)	<0.001	1.17 (1.06-1.29)	<0.001	1.37 (1.17-1.60)	<0.001
** Distant**								
LCNEC	Reference	—	Reference	—	Reference	—	Reference	—
SCLC	1.16 (1.06-1.28)	0.002	1.14 (1.03-1.25)	0.009	1.17 (1.06-1.29)	0.001	1.13 (1.03-1.25)	0.013
**Surgery**								
** No**								
LCNEC	Reference	—	Not applicable		Reference	—	Not applicable	
SCLC	1.06 (0.98-1.16)	0.155	Not applicable		1.06 (0.97-1.16)	0.216	Not applicable	
** Yes**								
LCNEC	Reference	—	Reference	—	Reference	—	Reference	—
SCLC	1.39 (1.21-1.59)	<0.001	1.31 (1.12-1.53)	0.001	1.55 (1.33-1.81)	<0.001	1.39 (1.17-1.65)	<0.001

Abbreviations: LCNEC, large cell neuroendocrine carcinoma; SCLC, small cell lung carcinoma; SEER, Surveillance Epidemiology and End Results database; HR, hazard ratio; CI, confidence interval.

**Table 4:** Clinicopathological characteristics of high-grade LCNEC and SCLC patients without surgery in 1:1 matched group

	Before the match			After the match		
	LCNEC N=620 (%)	SCLC N=17374 (%)	*P* value^a^	LCNEC N=620 (%)	SCLC N=620 (%)	*P* value
**Age**			0.431			0.16
<60	160 (25.8)	4340 (25.0)		160 (25.8)	163 (26.3)	
60-79	219 (35.3)	5835 (33.6)		219 (35.3)	189 (30.5)	
≥80	241 (38.9)	7199 (41.4)		241 (38.9)	268 (43.2)	
**Sex**			<0.001			0.954
Female	254 (41.0)	8718 (50.2)		254 (41.0)	253 (40.8)	
Male	366 (59.0)	8656 (49.8)		366 (59.0)	367 (59.2)	
**Race**			0.001			0.705
White	512 (82.6)	15266 (87.9)		512 (82.6)	517 (83.4)	
Black	79 (12.7)	1513 (8.7)		79 (12.7)	78 (12.6)	
Others^b^	27 (4.4)	580 (3.3)		27 (4.4)	25 (4.0)	
Unknown	2 (0.3)	15 (0.1)		2 (0.3)	0 (0.0)	
**Marital status**			0.562			0.072
Married	301 (48.5)	8744 (50.3)		301 (48.5)	341 (55.0)	
Not married^c^	296 (47.7)	8072 (46.5)		296 (47.7)	257 (41.5)	
Unknown	23 (3.7)	558 (3.2)		23 (3.7)	22 (3.5)	
**Laterality**			0.004			0.964
Left	231 (37.3)	7012 (40.4)		231 (37.3)	232 (37.4)	
Right	350 (56.5)	9560 (55.0)		350 (56.5)	352 (56.8)	
Bilateral	16 (2.6)	170 (1.0)		16 (2.6)	13 (2.1)	
Unknown	23 (3.7)	632 (3.6)		23 (3.7)	23 (3.7)	
**SEER stage**			0.426			0.28
Localized	38 (6.1)	909 (5.2)		38 (6.1)	54 (8.7)	
Regional	130 (21.0)	4011 (23.1)		130 (21.0)	139 (22.4)	
Distant	441 (71.1)	12069 (69.5)		441 (71.1)	416 (67.1)	
Unknown	11 (1.8)	385 (2.2)		11 (1.8)	11 (1.8)	
**Tumor size (cm)**			<0.001			1.000
≤3	136 (21.9)	2931 (16.9)		136 (21.9)	138 (22.3)	
3-5	115 (18.5)	3094 (17.8)		115 (18.5)	114 (18.4)	
5-7	91 (14.7)	2140 (12.3)		91 (14.7)	91 (14.7)	
>7	104 (16.8)	2407 (13.9)		104 (16.8)	102 (16.5)	
Unknown	174 (28.1)	6802 (39.2)		174 (28.1)	175 (28.2)	
**Nodal status**			<0.001			0.954
No	146 (23.5)	2668 (15.4)		146 (23.5)	150 (24.2)	
Yes	419 (67.6)	12531 (72.1)		419 (67.6)	417 (67.3)	
Unknown	55 (8.9)	2175 (12.5)		55 (8.9)	53 (8.5)	
**Radiation**			0.176			0.078
No/ Unknown	314 (50.6)	9285 (53.4)		314 (50.6)	346 (55.8)	
Yes	306 (49.4)	8089 (46.6)		306 (49.4)	274 (44.2)	
**Chemotherapy**			<0.001			0.953
No/ Unknown	234 (37.7)	5380 (31.0)		234 (37.7)	232 (37.4)	
Yes	386 (62.3)	11994 (69.0)		386 (62.3)	388 (62.6)	

Abbreviations: LCNEC, large cell neuroendocrine carcinoma; SCLC, small cell lung carcinoma; SEER, Surveillance Epidemiology and End Results database. ^a^ *P* value between high-grade LCNEC and SCLC was calculated by chi-square test. ^b^ Others included American Indian/Alaskan native, and Asian/Pacific islander. ^c^ Not married included separated, single (never married), divorced, unmarried or domestic partner and widowed.

## Abbreviations

AC: atypical carcinoid
CSS: cancer-specific survival
LCNEC: large-cell neuroendocrine tumor
NET: neuroendocrine tumor
NSCLC: non-small cell neuroendocrine tumor
OLC: other large-cell carcinoma
OS: overall survival
PSM: propensity score matching
SCLC: small-cell neuroendocrine tumor
SEER: Surveillance, Epidemiology, and End Results program
TC: typical carcinoid
