# Supplementary material for: Comparative study of large cell neuroendocrine carcinoma and small cell lung carcinoma in high-grade neuroendocrine tumors of the lung: a large population-based study
Source: J Cancer. 2019 Jul 10;10(18):4226–36. doi: 10.7150/jca.33367 (PMC6691699; doi:10.7150/jca.33367)
Supplement: Supplementary file 1 — Supplementary figure and table. [file jcav10p4226s1.pdf]

Supplementary Table 1. Univariate Cox proportional hazards regression model analysis of overall survival and cancer-special survival in high-grade LCNEC and SCLC patients

	Overall survival		Cancer-special survival	
	HR (95% CI)	*P* value	HR (95% CI)	*P* value
**Age**				
<60	Reference	—	Reference	—
60-79	1.18 (1.14-1.23)	<0.001	1.16 (1.11-1.21)	<0.001
≥80	1.59 (1.53-1.65)	<0.001	1.52 (1.46-1.58)	<0.001
**Sex**				
Female	Reference	—	Reference	—
Male	1.15 (1.11-1.18)	<0.001	1.14 (1.10-1.17)	<0.001
**Race**				
White	Reference	—	Reference	—
Black	0.98 (0.93-1.03)	0.492	0.98 (0.93-1.03)	0.433
Others[a]	0.98 (0.90-1.06)	0.541	0.94 (0.86-1.02)	0.152
Unknown	1.00 (0.58-1.73)	0.993	0.99 (0.56-1.74)	0.973
**Marital status**				
Married	Reference	—	Reference	—
Not married[b]	1.13 (1.10-1.16)	<0.001	1.11 (1.07-1.14)	<0.001
Unknown	1.14 (1.05-1.25)	0.002	1.13 (1.04-1.24)	0.006
**Laterality**				
Left	Reference	—	Reference	—
Right	1.00 (0.97-1.03)	0.818	1.00 (0.97-1.04)	0.780
Bilateral	1.67 (1.44-1.93)	<0.001	1.74 (1.50-2.02)	<0.001
Unknown	1.21 (1.12-1.31)	<0.001	1.20 (1.10-1.31)	<0.001
**SEER stage**				
Localized	Reference	—	Reference	—
Regional	1.44 (1.35-1.54)	<0.001	1.58 (1.47-1.70)	<0.001
Distant	2.90 (2.72-3.09)	<0.001	3.30 (3.08-3.54)	<0.001
Unknown	1.98 (1.77-2.22)	<0.001	2.22 (1.97-2.50)	<0.001
**Tumor size (cm)**				
≤3	Reference	—	Reference	—
3-5	1.26 (1.20-1.32)	<0.001	1.29 (1.22-1.36)	<0.001
5-7	1.35 (1.27-1.42)	<0.001	1.39 (1.31-1.47)	<0.001
>7	1.46 (1.39-1.54)	<0.001	1.51 (1.43-1.60)	<0.001
Unknown	1.69 (1.62-1.76)	<0.001	1.76 (1.69-1.84)	<0.001
**Nodal status**				
No	Reference	—	Reference	—
Yes	1.51 (1.45-1.57)	<0.001	1.58 (1.52-1.65)	<0.001

Unknown	1.70 (1.61-1.80)	<0.001	1.80 (1.69-1.90)	<0.001
**Surgery**				
No	Reference	—	Reference	—
Yes	0.35 (0.33-0.38)	<0.001	0.32 (0.30-0.35)	<0.001
Unknown	0.93 (0.79-1.09)	0.361	0.89 (0.75-1.06)	0.186
**Radiation**				
No/ Unknown	Reference	—	Reference	—
Yes	0.59 (0.58-0.61)	<0.001	0.60 (0.58-0.62)	<0.001
**Chemotherapy**				
No/ Unknown	Reference	—	Reference	—
Yes	0.49 (0.47-0.50)	<0.001	0.49 (0.48-0.51)	<0.001
**Histological type**				
LCNEC	Reference	—	Reference	—
SCLC	1.86 (1.74-1.99)	<0.001	1.95 (1.82-2.10)	<0.001

Abbreviations: LCNEC, large cell neuroendocrine carcinoma; SCLC, small cell lung carcinoma; SEER, Surveillance Epidemiology and End Results database; HR, hazard ratio; CI, confidence interval.

[a] Others included American Indian/Alaskan native, and Asian/Pacific islander.

[b] Not married included separated, single (never married), divorced, unmarried or domestic partner and widowed.

Supplementary Figure 1. OS and CSS for the high-grade LCNEC and SCLC patients in the non-surgery subgroup using Kaplan-Meier analysis and log-rank test. (A) OS in subgroup treated without surgery: LCNEC vs. SCLC, P = 0.16; (B) CSS in subgroup treated without surgery: LCNEC vs. SCLC, P = 0.06. Abbreviations: LCNEC, large cell neuroendocrine carcinoma; SCLC, small cell lung carcinoma; OS, overall survival; CSS, cancer-specific survival.

A

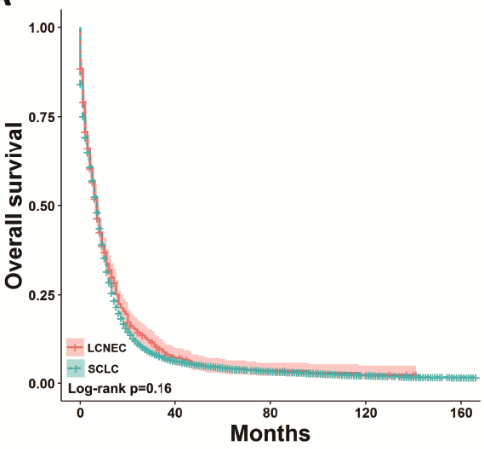

OS	1-year	2-year	3-year
LCNEC	31.9%	14.3%	8.1%
SCLC	28.5%	11.3%	7.0%

B

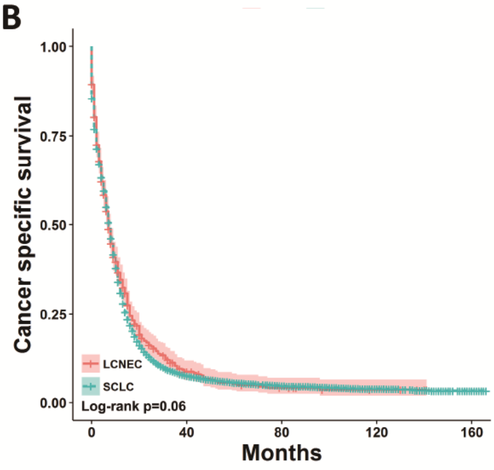

CSS	1-year	2-year	3-year
LCNEC	34.6%	16.1%	9.5%
SCLC	30.9%	12.8%	8.3%
